# Association among cytokine profiles of innate and adaptive immune responses and clinical-virological features in untreated patients with chronic hepatitis B

**DOI:** 10.1186/s12879-020-05233-x

**Published:** 2020-07-14

**Authors:** Yurong Gu, Yifan Lian, Qiaolan Zheng, Zexuan Huang, Lin Gu, Yanhua Bi, Jing Li, Yanlin Huang, Yuankai Wu, Lubiao Chen, Yuehua Huang

**Affiliations:** 1 Department of Infectious Diseases, the Third Affiliated Hospital of Sun Yat-sen University, 600 Tian He Rd, Guangzhou, 510630 China; 2 Guangdong Provincial Key Laboratory of Liver Disease Research, The Third Affiliated Hospital of Sun Yat-sen University, 600 Tian He Rd, Guangzhou, 510630 China; 3 Journal Center, The Third Affiliated Hospital of Sun Yat-sen University, 600 Tian He Rd, Guangzhou, 510630 China

**Keywords:** Chronic hepatitis B (CHB), Cytokines, Innate immunity, Adaptive immunity, Regression analysis

## Abstract

**Background:**

Complete clearance of intracellular viruses depends on effector cells of innate and adaptive immune systems. This study aimed to identify the relationships among antiviral cytokines produced by natural killer (NK) and T cells and clinical-virological characteristics in untreated chronic hepatitis B (CHB) patients.

**Methods:**

We measured antiviral cytokines interferon-gamma (IFN-γ), tumor necrosis factor-alpha (TNF-α), and interleukin-2 (IL-2) produced by T, NK and natural killer T (NKT) cells, respectively, in a cohort with chronic hepatitis B virus (HBV) infection (CHB). We also correlated these cytokines with clinical-virological characteristics using a linear regression model.

**Results:**

levels of IFN-γ^+^ and TNF-α^+^ CD4^+^ and CD8^+^ T cells were significantly higher in immune active (IA) phase than in other phases. Immune tolerant (IT) patients showed the lowest expression of IFN-γ by NK and NKT cells, and TNF-α by NK cells. IFN-γ^+^, TNF-α^+^ and IL-2^+^ CD4^+^ and CD8^+^ T cells frequencies were similar between IA and gray zone (GZ) phases. Principal component analysis based on cytokines confirmed that most IT patients significantly differed from inactive carriers (IC) and IA patients, while GZ patients were widely scattered. Multivariate analysis showed both T and NK cells producing IFN-γ and TNF-α, but not IL-2, had significant association with serum alanine aminotransferase (ALT). Moreover, IFN-γ^+^ NKT cells were associated with HBV DNA, while IFN-γ^+^ CD4^+^ and CD8^+^ T cells were correlated with age.

**Conclusion:**

HBV clinical phases are characterized by distinct cytokine signatures, which showed relationship to viral features in these untreated CHB patients.

## Background

Chronic infection with hepatitis B virus (HBV) is estimated to affect more than 240 million people worldwide, leading to 620,000 deaths per year [1, 2]. Although the host attempts to prevent and clean HBV infection with minimal damage to itself, the virus utilizes many strategies to escape from the host surveillance [3, 4]. At first, HBV must recognize and bind to specific receptors, and then enter the liver cells and migrate into the nucleus. In the nucleus, its genome is transcribed and translated to assemble and secrete new virions out of the infected cells, so HBV can spread to other susceptible cells [5–7]. In acute HBV infection, the host recognizes the virus and efficiently clears it as quickly as possible by a protective immune response [8–10]. Both of the innate and adaptive immunity play a critical role in fighting against HBV [11].

Due to the nature of HBV, the host may be triggered by the virus to produce antiviral cytokines that inhibit the HBV life cycle, to limit the spread of the infection [12]. Innate immune cells, including natural killer (NK) cells, granulocytes and natural killer T (NKT) cells, constitute the first line of defense. Although the innate and adaptive immune effector cells can directly destroy the infected liver cells, much of the antiviral ability of these cells results from production of antiviral cytokines at the site of infection, such as interferon-gamma (IFN-γ) and tumor necrosis factor-alpha (TNF-α) [13–15]. These antiviral cytokines can clear HBV from infected liver cells in a noncytopathological way, or indirectly control HBV infections, by enhancing the antigen-presenting process, promoting HBV epitope display at the surface of infected cells and regulating the immune response [3, 16]. Therefore, it is not surprising that the HBV proteins have the potential ability to inhibit the activity of these antiviral cytokines [17–20].

IFN-γ and TNF-α play an important role in HBV infection control in several ways. First, they can recruit and activate T cells, NK cells and macrophages to perform their functions, including producing antiviral and immunoregulatory monokines and cytokines. Second, they can induce T cells toward developing antiviral effector functions for effective control of HBV infection. Third, they can upregulate major histocompatibility complex (MHC) expression on infected liver cells, and then promote antigen processing and presentation. Finally, they can perform direct antiviral functions. In addition to IFN-γ and TNF-α, interleukin-2 (IL-2) is also an antiviral cytokine and can regulate the cellular immunity during HBV infection.

Although numerous studies have shown a host-virus relationship in CHB infection, few studies have attempted to test associations among innate immunity and T cell-derived antiviral cytokines and clinical-virological factors in a treatment-naïve CHB cohort. In this study, we measured antiviral cytokines, including IFN-γ, TNF-α and IL-2 produced by T cells, NK cells and NKT cells, respectively, in treatment-naïve CHB patients with different disease phases and analyzed the correlations between these cytokines and clinical characteristics.

## Methods

### Subjects

Adult CHB patients in the viral hepatitis clinic of the Third Affiliated Hospital of Sun Yat-sen University were recruited. Patients who received antiviral treatment within the previous 6 months; with end-stage liver insufficiency, cirrhosis, and malignancies; with autoimmune disorders or, immunosuppressive treatment; and with human immunodeficiency virus, hepatitis C virus, or hepatitis D virus coinfection, were excluded. The study was approved by the Institutional Ethical Board of Sun Yat-sen University and carried out conforming to the Code of Ethics of the World Medical Association (Declaration of Helsinki) for experiments involving humans. Written informed consent was obtained from all patients.

Of 244 eligible patients, 15 were excluded because of missing values and 229 patients were analyzed. Classification of the patients in this work was in accordance with published international CHB treatment guidelines, as follows: (1) immune tolerant (IT) phase: normal ALT level, elevated HBV DNA load, typically > 1 million IU mL-1, and hepatitis B e antigen (HBeAg) positive; (2) Immune active (IA) phase: elevated ALT level, HBeAg positive and HBV DNA > 20,000 IU mL-1, or HBeAg negative and HBV DNA > 2000 IU mL-1; (3) Inactive carriers (IC): normal ALT level, antibody to hepatitis B e antigen (HBeAb) positive, and low HBV DNA level; and (4); gray zones (GZ): ALT and HBV DNA levels did not fall into the same traditionally characterized phases [21]. Blood was also obtained from age-matched healthy controls (*n* = 17). Information on the demographics, HBV markers (HBeAg, HBV DNA, hepatitis B surface antigen [HBsAg]), HBV genotypes and liver function is listed in Table 1.

**Table 1:** Clinical-virological characteristics of patients included in the study

Characteristics	IT (*n* = 17)	IA (*n* = 120)	IC (*n* = 20)	GZ (*n* = 72)	HC (n = 17)	*P* value
Age, years, median (quartile)	25 (24, 26)	29 (25, 33.25)	32 (28.75, 37)	31.5 (26, 38.25)	27 (25.5, 36)	< 0.001
Gender						0.238
Male, n (%)	12 (70.6)	77 (64.2)	17 (85)	55 (76.4)	12 (70.6)	
Female, n (%)	5 (29.4)	43 (35.8)	3 (15)	17 (23.6)	5 (29.4)	
ALT, U/L, median (quartile)	20.6 (18.3, 22.4)	20.8 (19.1, 22.5)	22.3 (21.6, 23.8)	21.484 (19.8, 23.4)	16 (12.5, 22.0))	< 0.001
Fibroscan, Kpa median (quartile)	4.9 (4.2, 5.4)	5.3 (4.3, 6.5)	4.4 (4.0, 5.3)	4.8 (4.4, 5.4)	4.6 (4.0 ~ 5.1)	0.016
HBV DNA, Log IU/ml, median (quartile)	8.2 (8.2, 8.2)	7.7 (5.0, 8.2)	2.2 (1.6, 3.1)	3.3 (2.1, 4.3)		< 0.001
HBeAg status						< 0.001
Negative, n (%)	0 (0)	41 (34)	20 (100)	62 (86)		
Positive, n (%)	17 (100)	78 (65)	0 (0)	10 (14)		
Missing, n (%)	0 (0)	1 (1)	0 (0)	0 (0)		
HBeAb status						< 0.001
Negative, n (%)	17 (100)	70 (58.3)	1 (5)	13 (18.1)		
Positive, n (%)	0 (0)	47 (39.2)	19 (95)	59 (81.9)		
Missing, n (%)	0 (0)	3 (2.5)	0 (0)	0 (0)		
qHBsAg, Log IU/ml, median (quartile)	4.6 (4.5, 4.7)	4.0 (3.3, 4.7)	2.9 (2.0, 3.2)	3.2 (2.3, 3.6)		< 0.001
HBsAb status						< 0.001
Negative, n (%)	15 (88)	106 (88)	20 (100)	67 (93)		
Positive, n (%)	2 (12)	14 (12)	0 (0)	5 (7)		
HBV genotype						< 0.001
C, n(%)	2 (12)	30 (25)	3 (15)	15 (21)		
B, n (%)	12 (71)	74 (62)	8 (40)	27 (38)		
N, n (%)	0 (0)	2 (2)	9 (45)	23 (32)		
O, n (%)	1 (6)	8 (1)	0 (0)	23 (32)		
Missing, n(%)	2 (11)	6 (5)	0 (0)	1 (1)		

HBV genotype: Other included C + D, B + D, B + C, D; N, not detected

*IT* immune tolerant, *IA* immune active, *IC* inactive carrier, *GZ* gray zones, *ALT* alanine aminotransferase, *HBeAb* antibody to HBV e antigen, *HBeAg,* HBV e antigen, *HBsAb* antibody to hepatitis B surface antigen, *qHBsAg* quantitative hepatitis B surface antigen;

### Cell surface marker staining and flow cytometry analysis

According to the manufacturer’s instructions, peripheral blood mononuclear cells (PBMCs) were isolated using Ficoll density gradients. PBMCs were stimulated with Leukocyte Activation Cocktail (BD Pharmingen, San Diego, CA) at 37 °C for 4 h prior to intracellular staining using the manufacturer’s staining protocol. PBMCs were stained for surface markers, fixed, permeabilized with IntraPreReagent (Beckman Coulter, Fullerton, CA), and then stained with antibodies for intracellular markers. Anti-human mAbs against APC-CD4, FITC-CD56, FITC-IFN-γ, PE-CF594-CD3, PE-IL-2, PE-TNF-α, and V450-CD8, with controls, were purchased from BD Biosciences (San Jose, CA, USA). Data were acquired on a Gallios instrument (Beckman Coulter, Brea, CA) and analyzed with FlowJo software (Ashland, OR).

### Clinical and serologic parameters

Upon recruitment, patient serum was tested for hepatitis B surface antibody (HBsAb), HBeAg and HBeAb, using commercial kits (Abbott Laboratories, North Chicago, IL). Quantitative hepatitis B surface antigen (qHBsAg) was measured by Elecsys HBsAg II Quant reagent kits (Roche Diagnostics, Indianapolis, IN). Serum HBV DNA levels were measured by Roche COBAS Ampliprep/COBAS TaqMan HBV Test v2.0 (dynamic range from 20 to 1.7E + 08 IU mL-1, Roche Molecular Diagnostics, Branchburg, NJ).). Six HBV genotypes (a-f) were assed by direct sequencing. The levels of fibrosis were defined by liver stiffness measurement (Fibroscan, Echosens, Paris, France).

### Statistical analysis

Comparisons between the two patient groups were performed using the Mann-Whitney test for continuous variables and the χ2 test for categorical variables. We explored the association between continuous variables using a linear regression model, Pearson correlation or Spearman correlation. For the cluster analysis, we used principal component analysis to separate the samples into four clusters. All the other statistical tests were performed using R software version 3.2.2. Statistical significance was set to 0.05.

## Results

### Baseline characteristics of the study population

To study viral and immune correlations in the different CHB disease phases, we carefully selected a homogeneous cohort of untreated chronic HBV infected patients without any other comorbidities, attending our outpatient clinic. To rule out the impact of advanced liver fibrosis on any identified immune parameter, patients with advanced fibrosis (F2 fibrosis or higher) were excluded. As is typical for the natural history of CHB patients, IT patients were youngest among the patient cohort. Owing to the stringent definition criteria, differences in age, ALT and HBV DNA levels were observed between clinical phases. Unlike some recent reports, the qHBsAg level in this study was higher in IT patients than in GZ patients [22, 23] (Table 1).

### Cytokine profiles in CHB patients with different stages of disease

**Fig. 1:**
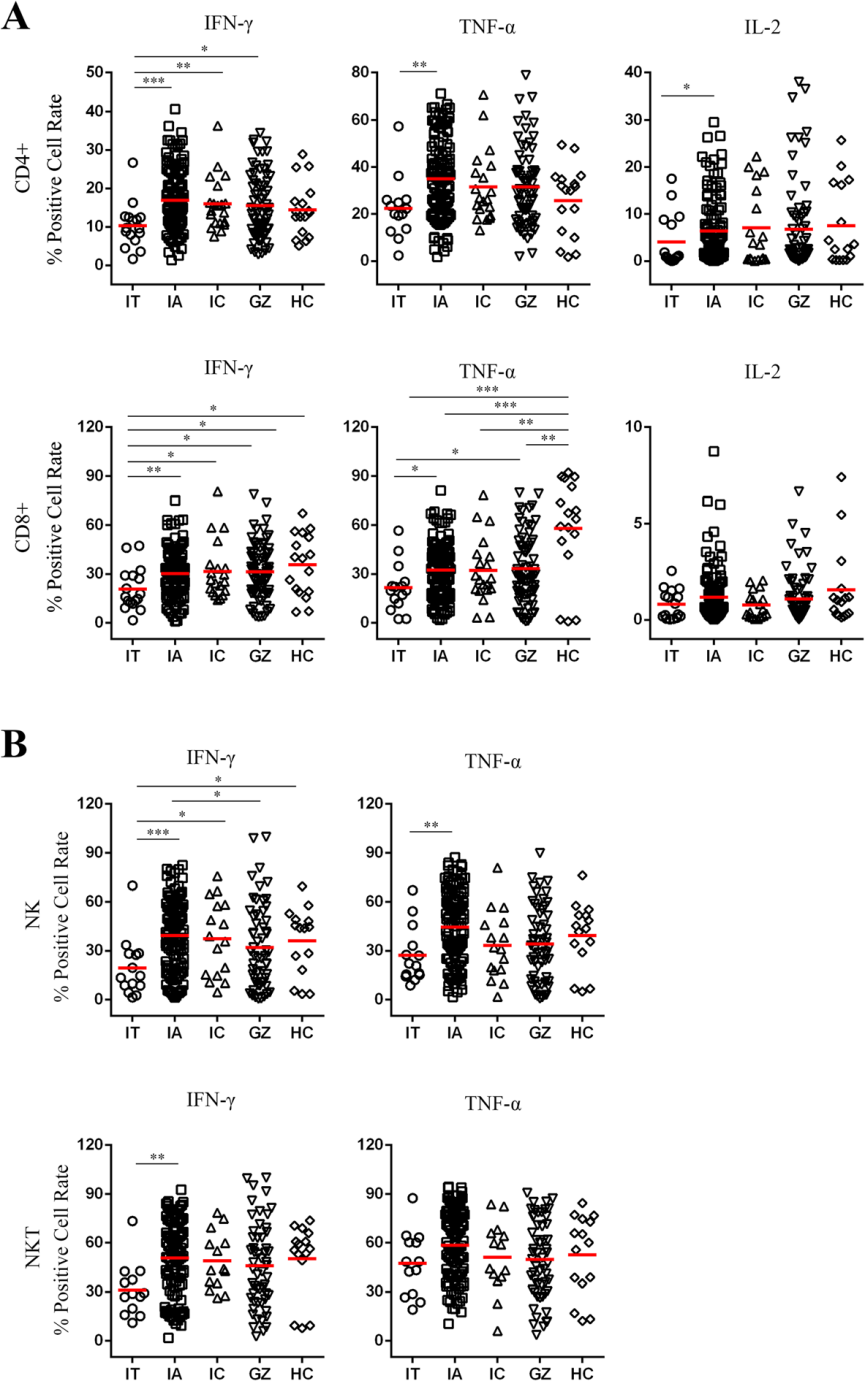
Cytokine profiles of innate and adaptive immune responses from naïve CHB patients. **a** Expression of cytokines IFN-γ^+^, TNF-α^+^ and IL-2^+^ by CD4^+^ and CD8^+^ T cells derived from the indicated patient groups was measured. The levels were compared among patients in the IT, IA, GZ and IC phases, and those of healthy controls. **b** Expression levels of cytokines in IFN-γ^+^, TNF-α^+^ NK and NKT cells derived from the indicated patient groups were measured. The levels were compared among patients in the IT, IA, GZ and IC phases, and healthy controls. Differences between two patient groups were evaluated by the Mann-Whitney test. Data are presented as the median indicated by a red line. *P <  0.05, **P <  0.01, ***P <  0.001

To investigate whether CHB patients beyond current treatment criteria are characterized by a state of defective antiviral response, we analyzed the expression profiles of three major effector cytokines, IFN-γ, TNF-α and IL-2, produced by innate and adaptive immunity. The representative dot plots and gating strategies of flow cytometry analysis for T cell-, NK cell- and NKT cell-derived cytokines are shown in Supplementary Figure 1. We first analyzed their T cell-derived cytokine profiles and compared them with those in healthy controls. The frequency of IFN-γ^+^ CD4+ T cells was significantly higher in the IA, IC and GZ groups than in the IT group. The frequency of IFN-γ^+^ CD8+ T cells was significantly higher in the IA, IC, GZ and HC group than in the IT group. Moreover, the frequency of TNF-α^+^ CD8+ T cells was higher in the GZ and IA groups than in the IT group while frequency of TNF-α^+^ CD8+ T cells by HC group was higher than that in the GZ, IC, IA and IT groups. Both the frequencies of TNF-α^+^ and IL-2^+^ CD4+ T cells were significantly higher in IA patients than in IT patients (Fig. 1a).

We also measured the frequencies of cytokines produced by NK and NKT cells in the current CHB cohort. As expected, statistically significant differences were observed in the frequencies of NK and NKT cells secreting IFN-γ, with progressive decreasing levels from IA, IC, GZ, and IT patients. The differences of the frequencies of IFN-γ produced by NKT cells, IFN-γ and TNF-α produced by NKT and NK cells, respectively, between the patients in IA and GZ phases were not statistically significant. However, the frequencies of IFN-γ produced by NKT and NK cells and TNF-α produced by NK cells of patients in the IA phase were all higher than those of patients in the IT phase (*P* = 0.004 and 0.0008 for IFN-γ^+^ NKT and NK cells in IA vs IT; P = 0.004 for TNF-α^+^ NK cells in IA vs IT, respectively, Fig. 1b).

Taken together, these results indicate that a certain number of CHB patients beyond the current treatment guidelines, particularly, patients in the GZ phase, still produce antiviral cytokines.

### Distribution of distinct cytokine profiles in CHB patients with different disease phases

**Fig. 2:**
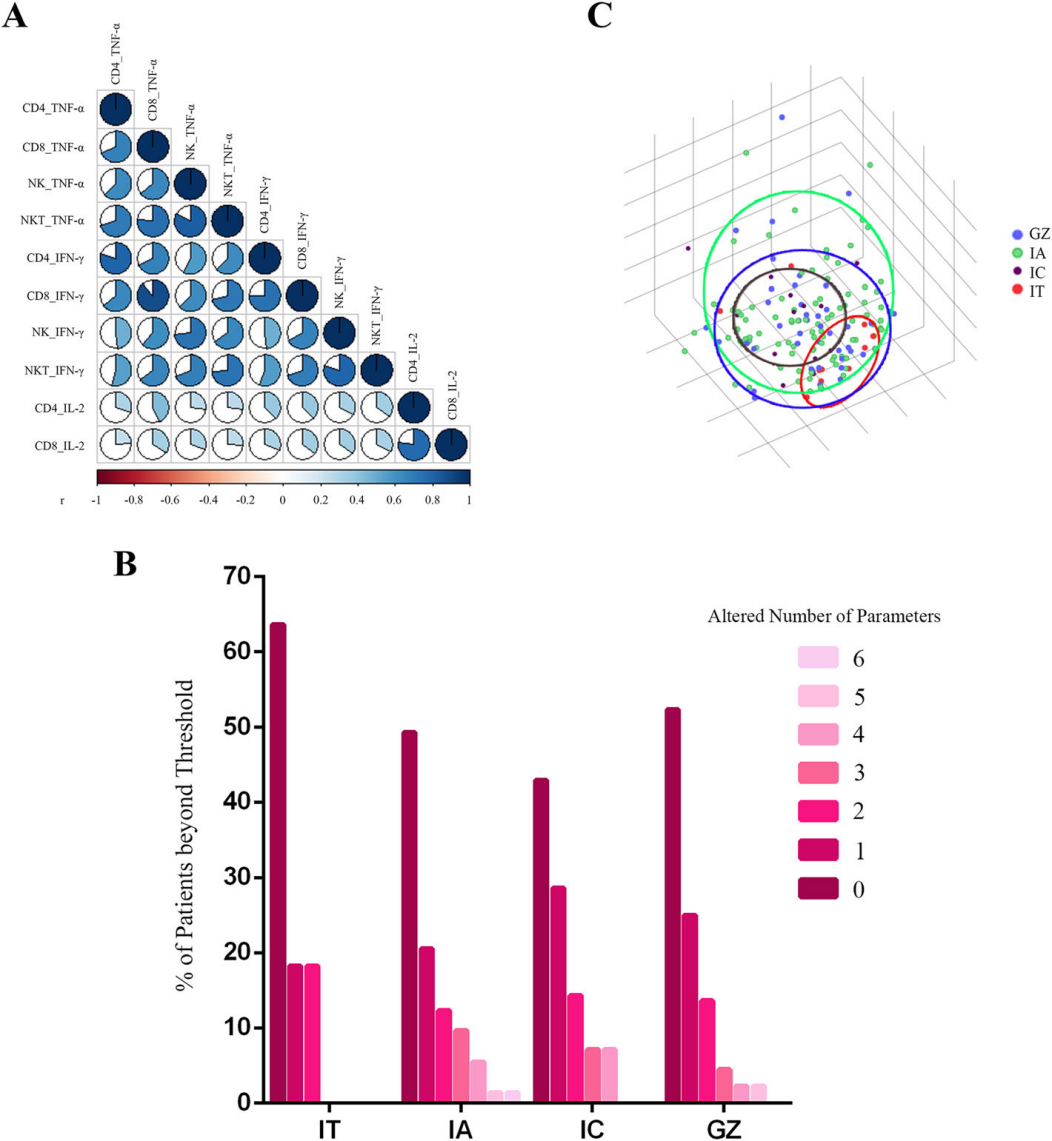
Distribution of distinct cytokine profiles in CHB patients with different disease phases. **a** Correlations among ten cytokines, IFN-γ^+^, TNF-α^+^ and IL-2^+^ CD4^+^ and CD8^+^ T cells and IFN-γ^+^ and TNF-α^+^ NK and NKT cells, were measured by Spearman correlation. P <  0.05 was colored, and pseudocolors indicate correlation levels from negative (− 1) to positive (1), ranging from a weak (white) to strong (red or blue) association strength. **b** Summary of percentages of patients in the IT, IA, IC and GZ phases grouped by altered applicable parameters. There were individuals with up to 6 altered applicable parameters in the IA group, and the number in the IC was 4, IT only 2, GZ however, 5 representing an intermediate state. **c** Representative image of principal component analysis based on CD4_TNF-α^+^, CD8_IL-2^+^, NK_IFN-γ^+^. Each circle represents a single patient and different colors identify different patient categories (IT, IC, IA and GZ patients in red, brown, green and blue, respectively)

Because clinical-virological features from patients with CHB were associated with TNF-α^+^, IFN-γ^+^ and IL-2^+^ T cells, and TNF-α^+^ and IFN-γ^+^ NK cells, we then assessed whether their combined evaluation could be used to identify the maturation of an efficient antiviral response to therapy in individual treatment-naïve CHB patients. We first investigated the correlation among the current 3 pairs of T-cell subset cytokines and 2 pairs of NK and NKT cell cytokines. The overall correlation among these 10 cytokines is shown in Fig. 2a. After correlation analysis, the expression levels of 6 cytokines (CD4_IFN-γ^+^, CD4_IL-2^+^, CD8_TNF-α^+^, CD8_IL-2^+^, NK_IFN-γ^+^, NKT_TNF-α^+^) were selected to construct an IA-similar cytokine profiles. The assumption was that acquisition of IA-similar cytokine profiles could reflect a vigorous response to antiviral therapy. A threshold was thus established as shown by the mean value found in IA patients plus one standard deviation for the above selected parameters, and calculation of their expressions in individual patients was conducted to compare with each matched threshold. Individual cytokine distribution profiles were distinguished according to the altered number of applicable parameters beyond the threshold for all patients. A profile with all the applicable parameters altered was assumed to reflect an active immune response to therapy whereas a profile with no applicable parameters altered was predicted to be associated with an awakening response to therapy. IA and IC with an active immune response to HBV showed a prevalent expression of more inflammatory patterns with 6 and 4 altered applicable parameters respectively. In contrast, IT patients showed an immune depletive pattern with only 2 altered application parameters. GZ patients instead showed an intermediate behavior with 5 altered applicable parameters, as a likely result of the transition from an immune depletive to an inflammatory pattern of typical IA patients (Fig. 2b). Based on Spearman’s rank correlation analysis, CD4_TNF-α^+^, CD8_IL-2^+^ and NK_IFN-γ^+^ were selected as the representative cytokines in the current CHB cohort for principal component analysis, which further confirmed that most IT patients significantly differed from IA and IC patients (red, blue and black circles, respectively), both of whom clustered homogeneously and an intermediate distribution was observed for GZ patients, who were widely scattered (green circles, Fig. 2c).

### Association among T cell-secreted cytokines and correlation with clinical-virological characteristics

Linear regression analysis was used to examine the association between T cell-produced cytokines and clinical-virological parameters. Univariate analysis revealed that a positive HBeAg result, and elevated levels of ALT and HBV DNA were associated with increased levels of CD4^+^ T cell-secreted TNF-α. Older age and higher ALT levels were associated with more proportion of IFN-γ^+^ CD4^+^ T cells, while IL-2^+^ CD4^+^T cells were linked to the increased HBV genotypes. After adjusting for other confounding factors, multivariate analysis revealed that both higher ALT levels and older age were significantly associated with increased IFN-γ^+^ CD4^+^ T cells, and gender, ALT and HBV genotype were significantly associated with TNF-α^+^ CD4^+^ T cells (Table 2).

**Table 2:** Factors associated with CD4+ T cells secreting cytokines in the univariate and multivariate linear regression analysis

Variable	CD4_IFN-γ				CD4_TNF-α				CD4_IL-2			
	Univariate (raw effects)		Multivariate (adjusted effects)		Univariate (raw effects)		Multivariate (adjusted effects)		Univariate (raw effects)		Multivariate (adjusted effects)	
	B (95% CI)	*P*	Adjusted B (95% CI)	Adjusted *P*	B (95% CI)	*P*	Adjusted B (95% CI)	Adjusted *P*	B (95% CI)	*P*	Adjusted B (95% CI)	Adjusted *P*
Age (≥ 25 vs < 25 years)	4.26 (1.15, 7.36)	**0.008**	4.09 (0.94, 7.24)	**0.011**	5.47 (−0.99, 11.93)	0.097	5.76 (− 0.52, 12.04)	0.072	0.11 (− 0.36, 0.58)	0.635	0.09 (− 0.42, 0.59)	0.729
Gender (Male ve Female)	2.35 (− 0.6, 5.3)	0.118	2.38 (− 0.55, 5.31)	0.110	5.48 (− 0.55, 11.52)	0.075	6.42 (0.57, 12.26)	**0.032**	0.22 (−0.22, 0.65)	0.334	0.13 (−0.34, 0.6)	0.586
Family History (Yes vs No)	0.56 (−2.14, 3.26)	0.684	0.55 (− 2.41, 3.51)	0.714	1.96 (−3.57, 7.48)	0.485	0.03 (−5.87, 5.94)	0.991	0.01 (−0.38, 0.41)	0.943	−0.03 (− 0.5, 0.44)	0.903
Vertical Transmission (Yes vs No)	−1.34 (−5, 2.33)	0.472	− 1.42 (− 5.41, 2.57)	0.483	2.05 (− 5.47, 9.57)	0.591	1.00 (−6.96, 8.97)	0.804	−0.01 (− 0.56, 0.53)	0.961	− 0.03 (− 0.67, 0.61)	0.931
Infection Time (≥ 20 vs < 20 years)	−0.2 (−4.01, 3.6)	0.917	−1.88 (− 5.74, 1.98)	0.336	0.85 (− 6.95, 8.64)	0.831	− 1.49 (−9.19, 6.2)	0.702	− 0.08 (− 0.65, 0.48)	0.769	−0.16 (− 0.77, 0.46)	0.616
Log HBsAg	0.60 (− 1.54, 2.75)	0.577	0.79 (− 1.35, 2.93)	0.466	0.004 (− 4.39–4.40)	0.999	0.42 (− 3.85, 4.68)	0.848	0.08 (− 0.24, 0.40)	0.621	0.01 (− 0.33, 0.36)	0.938
Log HBV DNA	0.09 (−0.45, 0.63)	0.741	−0.99 (− 2.09, 0.11)	0.078	1.45 (0.36–2.54)	**0.010**	−0.74 (− 2.94, 1.45)	0.505	0.26 (− 0.2, 0.72)	0.260	− 0.06 (− 0.24, 0.11)	0.483
HBeAg (Positive vs Negative)	1.18 (− 1.51, 3.87)	0.387	4.30 (− 0.10, 8.70)	0.055	7.18 (1.77, 12.58)	**0.010**	8.25 (−0.53, 17.03)	0.065	−0.22 (− 0.62, 0.17)	0.266	− 0.01 (− 0.72, 0.69)	0.969
ALT (< 2 ULN is the reference group)												
≥ 2 & < 5 ULN	6.16 (2.76, 9.56)	**0.000**	6.77 (3.08, 10.46)	**0.000**	14.41 (7.64, 21.19)	**0.000**	13.08 (5.72, 20.44)	**0.001**	−0.14 (− 0.67, 0.38)	0.594	− 0.05 (− 0.64, 0.54)	0.859
≥ 5ULN	3.89 (− 0.34, 8.12)	0.072	3.82 (− 0.79, 8.44)	0.104	13.2 (4.77, 21.64)	**0.002**	9.08 (−0.12, 18.29)	0.053	0.20 (−0.45, 0.86)	0.539	0.23 (−0.51, 0.97)	0.533
Genotype (Genotype C is the reference group)												
B	−0.84 (−4.2, 2.53)	0.623	−0.14 (−3.42, 3.13)	0.930	−4.03 (−10.79, 2.73)	0.241	−2.5 (−9.04, 4.04)	0.451	− 0.52 (− 1.02, − 0.03)	**0.039**	−0.49 (− 1.02, 0.03)	0.067
N	−0.85 (−5.43, 3.73)	0.715	− 2.39 (−7.83, 3.06)	0.388	− 9.19 (− 18.39, 0.01)	0.050	−6.88 (− 17.75, 3.99)	0.213	− 0.36 (− 1.04, 0.31)	0.288	− 0.59 (− 1.46, 0.28)	0.184
O	−5.27 (− 10.98, 0.45)	0.071	−3.46 (− 9.08, 2.17)	0.226	−16.2 (− 27.68, − 4.72)	0.006	−11.84 (− 23.06, − 0.63)	**0.039**	− 0.68 (− 1.52, 0.16)	0.113	− 0.64 (− 1.54, 0.26)	0.161

Significant values are shown in boldface. B: Unstandardized Coefficients. HBV genotype: Other included C + D, B + D, B + C, D; N, not detected

*ALT* alanine aminotransferase, *HBeAg* HBV e antigen, *HBsAg* hepatitis B surface antigen

Univariate analysis of the relationship between CD8+ T cell-derived cytokines and clinical-virological factors showed that age and ALT were associated with both TNF-α^+^ and IFN-γ^+^ CD8^+^ T cells, respectively. Multivariate analysis indicated that older age and high ALT were still associated with increased IFN-γ content, while only ALT was significantly related to increased TNF-α^+^ CD8^+^ T cell proportions. There was no statistically significant association between IL-2^+^ CD8^+^ T cells and viral parameters (Table 3).

**Table 3:** Factors associated with CD8+ T cells secreting cytokines in the univariate and multivariate linear regression analysis

Variable	CD8_IFN-γ				CD8_TNF-α				CD8_IL-2			
	Univariate (raw effects)		Multivariate (adjusted effects)		Univariate (raw effects)		Multivariate (adjusted effects)		Univariate (raw effects)	Multivariate (adjusted effects)		
	B (95% CI)	*P*	Adjusted B (95% CI)	Adjusted *P*	B (95% CI)	*P*	Adjusted B (95% CI)	Adjusted *P*	B (95% CI)	*P*	Adjusted B (95% CI)	Adjusted *P*
Age (≥ 25 vs < 25 years)	8.08 (1.79, 14.36)	**0.012**	6.92 (0.41, 13.43)	**0.037**	8.2 (1.04, 15.37)	**0.025**	6.89 (−0.59, 14.37)	0.071	0.11 (− 0.36, 0.58)	0.635	0.09 (− 0.42, 0.59)	0.729
Gender (Male ve Female)	5.25 (−0.69, 11.2)	0.083	3.36 (−2.7, 9.41)	0.275	5.41 (− 1.35, 12.17)	0.116	3.81 (−3.15, 10.77)	0.281	0.22 (−0.22, 0.65)	0.334	0.13 (−0.34, 0.6)	0.586
Family History (Yes vs No)	−1.42 (−6.86, 4.02)	0.607	−3.27 (−9.39, 2.86)	0.293	−0.56 (− 6.75, 5.62)	0.858	−2.22 (− 9.26, 4.82)	0.534	0.01 (− 0.38, 0.41)	0.943	−0.03 (− 0.5, 0.44)	0.903
Vertical Transmission (Yes vs No)	−0.84 (−8.24, 6.57)	0.824	1.82 (− 6.43, 10.08)	0.663	− 0.85 (− 9.26, 7.55)	0.841	0.72 (− 8.77, 10.2)	0.881	− 0.01 (− 0.56, 0.53)	0.961	−0.03 (− 0.67, 0.61)	0.931
Infection Time (≥ 20 vs < 20 years)	4.76 (− 2.88, 12.4)	0.220	0.97 (− 7, 8.94)	0.810	6.1 (− 2.56, 14.75)	0.166	2.69 (− 6.47, 11.86)	0.562	− 0.08 (− 0.65, 0.48)	0.769	− 0.16 (− 0.77, 0.46)	0.616
Log HBsAg	− 0.021 (− 0.033, − 0.009)	**0.001**	−0.2 (− 4.62, 4.23)	0.931	0.54 (− 4, 37, 5.44)	0.829	0.98 (− 4.11, 6.07)	0.704	0.08 (− 0.24, 0.40)	0.621	0.01 (− 0.33, 0.36)	0.938
Log HBV DNA	−0.054 (− 0.087, − 0.021)	**0.001**	−2.08 (− 4.35, 0.2)	0.073	−0.12 (− 1.37, 1.12)	0.849	−1.59 (− 4.2, 1.03)	0.232	0.26 (− 0.2, 0.72)	0.260	− 0.06 (− 0.24, 0.11)	0.483
HBeAg (Positive vs Negative)	−2.35 (− 7.78, 3.08)	0.394	3.87 (− 5.24, 12.97)	0.402	− 1.37 (− 7.54, 4.81)	0.662	2.98 (−7.48, 13.45)	0.574	− 0.22 (− 0.62, 0.17)	0.266	− 0.01 (− 0.72, 0.69)	0.969
ALT (< 2 ULN is the reference group)												
≥ 2 & < 5 ULN	10.78 (3.81, 17.75)	**0.003**	12.5 (4.87, 20.13)	**0.002**	12.08 (4.18, 19.99)	**0.003**	13.05 (4.29, 21.82)	**0.004**	−0.14 (− 0.67, 0.38)	0.594	− 0.05 (− 0.64, 0.54)	0.859
≥ 5ULN	2.77 (−5.9, 11.45)	0.528	4.93 (−4.61, 14.48)	0.309	5.47 (− 4.38, 15.31)	0.274	6.68 (−4.29, 17.65)	0.230	0.20 (−0.45, 0.86)	0.539	0.23 (−0.51, 0.97)	0.533
Genotype (Genotype C is the reference group)												
B	−3.57 (−10.39, 3.24)	0.302	−2.42 (−9.2, 4.36)	0.481	−6.27 (−16.8, 4.25)	0.240	− 3.21 (− 11, 4.58)	0.417	−0.52 (− 1.02, − 0.03)	**0.039**	− 0.49 (− 1.02, 0.03)	0.067
N	− 3.71 (− 12.99, 5.56)	0.430	− 10.05 (− 21.31, 1.22)	0.080	−8.65 (− 21.77, 4.47)	0.195	−10.2 (− 23.15, 2.74)	0.121	− 0.36 (− 1.04, 0.31)	0.288	−0.59 (− 1.46, 0.28)	0.184
O	− 8.44 (− 20.01, 3.13)	0.152	−7.3 (− 18.92, 4.32)	0.216	− 4.83 (− 12.56, 2.9)	0.219	− 6.66 (− 20.01, 6.7)	0.326	− 0.68 (− 1.52, 0.16)	0.113	− 0.64 (− 1.54, 0.26)	0.161

Significant values are shown in boldface. B: Unstandardized Coefficients. HBV genotype: Other included C + D, B + D, B + C, D; N, not detected

*ALT* alanine aminotransferase, *HBeAg* HBV e antigen, *HBsAg* hepatitis B surface antigen;

Therefore, either IFN-γ^+^ CD4^+^ or CD8^+^ T cells were significantly associated with older age and higher ALT, while TNF-α produced by these T cell subsets was associated with ALT.

### Association among NK and NKT cell-secreted cytokines and correlation with clinical-virological characteristics

Similarly, the linear regression analysis was used to examine the association between clinical-virological factors and NK or NKT cell-expressed cytokines in the current CHB cohort. Univariate analysis of the NK cell cytokine profiles showed that elevated frequencies of IFN-γ^+^ and TNF-α^+^ cells were correlated with higher ALT levels, while only TNF-α^+^ cells were also associated with HBV DNA. Multivariate analysis also showed similar results regarding the association between cytokines and ALT (Table 4). We also detected cytokines produced by a subset of T cells that express NK cell markers, NKT cells. The HBeAg, HBV DNA and genotypes were found to be associated with NKT cell-secreted TNF-α via univariate analysis. Both of univariate and multivariate analysis showed that ALT was associated with NKT cell-secreted TNF-α, and ALT and HBV DNA were associated with NKT cell-secreted IFN-γ (Table 5).

**Table 4:** Factors associated with NK cells secreting cytokines in the univariate and multivariate linear regression analysis

Variable	NK_IFN-γ				NK_TNF-α			
	Univariate (raw effects)		Multivariate (adjusted effects)		Univariate (raw effects)		Multivariate (adjusted effects)	
	B (95% CI)	*P*	Adjusted B (95% CI)	Adjusted *P*	B (95% CI)	*P*	Adjusted B (95% CI)	Adjusted *P*
Age (≥ 25 vs < 25 years)	3.36 (−5.44, 12.16)	0.452	3 (− 5.91, 11.9)	0.507	4.93 (−4.1, 13.97)	0.282	2.84 (−5.53, 11.2)	0.503
Gender (Male ve Female)	0.32 (−7.92, 8.56)	0.939	−1.23 (− 9.52, 7.06)	0.769	1.87 (− 6.61, 10.36)	0.663	1.68 (− 6.11, 9.47)	0.670
Family History (Yes vs No)	−0.82 (−8.3, 6.66)	0.829	−3.98 (−12.36, 4.4)	0.349	− 2.24 (− 9.93, 5.45)	0.566	−6.54 (− 14.41, 1.33)	0.103
Vertical Transmission (Yes vs No)	− 1.64 (− 11.81, 8.53)	0.750	0.51 (− 10.79, 11.8)	0.929	1.54 (− 8.92, 12.01)	0.771	4.82 (− 5.8, 15.43)	0.371
Infection Time (≥ 20 vs < 20 years)	6.21 (− 4.28, 16.7)	0.244	4.06 (− 6.85, 14.97)	0.463	6.95 (− 3.84, 17.74)	0.205	4.32 (−5.93, 14.57)	0.406
Log HBsAg	−0.66 (−6.60, 5.27)	0.825	− 0.72 (− 6.78, 5.33)	0.814	−3.73 (− 9.81, 2.35)	0.227	− 2.55 (− 8.24, 3.14)	0.376
Log HBV DNA	0.024 (− 0.001, 0.048)	0.058	−2.84 (− 5.95, 0.28)	0.074	2.02 (0.50, 3.53)	**0.009**	− 0.19 (− 3.11, 2.74)	0.899
HBeAg (Positive vs Negative)	2.61 (−4.85, 10.07)	0.490	8.29 (−4.17, 20.75)	0.190	7.08 (−0.53, 14.68)	0.068	1.38 (−10.32, 13.09)	0.816
ALT (< 2 ULN is the reference group)								
≥ 2 & < 5 ULN	16.73 (7.3, 26.15)	**0.001**	17.63 (7.19, 28.07)	**0.001**	25.61 (16.89, 34.33)	**0.000**	24.39 (14.58, 34.19)	**0.000**
≥ 5ULN	11.45 (−0.27, 23.18)	0.056	11.66 (−1.4, 24.72)	0.080	28.69 (17.84, 39.54)	**0.000**	27.64 (15.37, 39.91)	**0.000**
Genotype (Genotype C is the reference group)								
B	−6.57 (−15.74, 2.6)	0.159	−5.2 (− 14.47, 4.08)	0.270	−7.33 (− 16.87, 2.22)	0.132	−4.23 (− 12.94, 4.49)	0.339
N	− 10.05 (− 22.53, 2.43)	0.114	− 15.6 (−31.01, − 0.19)	**0.047**	−10.81 (− 23.8, 2.19)	0.102	− 5.2 (− 19.68, 9.28)	0.479
O	− 22.02 (− 37.59, − 6.46)	**0.006**	− 20.06 (− 35.96, − 4.15)	**0.014**	−15.64 (− 31.85, 0.57)	**0.058**	−9.13 (− 24.07, 5.81)	0.229

Significant values are shown in boldface. B: Unstandardized Coefficients. HBV genotype: Other included C + D, B + D, B + C, D; N, not detected

*ALT* alanine aminotransferase, *HBeAg* HBV e antigen, *HBsAg*, hepatitis B surface antigen;

**Table 5:** Factors associated with NKT cells secreting cytokines in the univariate and multivariate linear regression analysis

Variable	NKT_IFN-γ				NKT_TNF-α			
	Univariate (raw effects)		Multivariate (adjusted effects)		Univariate (raw effects)		Multivariate (adjusted effects)	
	B (95% CI)	*P*	Adjusted B (95% CI)	Adjusted *P*	B (95% CI)	*P*	Adjusted B (95% CI)	Adjusted *P*
Age (≥ 25 vs < 25 years)	8.58 (− 0.42, 17.57)	0.061	7.6 (−1.6, 16.79)	0.105	5.92 (− 2.86, 14.7)	0.185	5.08 (−3.41, 13.57)	0.238
Gender (Male ve Female)	3.26 (−5.24, 11.76)	0.450	1.42 (−7.14, 9.98)	0.743	4.94 (−3.28, 13.16)	0.237	5.15 (−2.75, 13.05)	0.200
Family History (Yes vs No)	−1.76 (−9.48, 5.96)	0.653	−4.77 (−13.42, 3.88)	0.277	0.84 (−6.65, 8.33)	0.824	−3.09 (−11.07, 4.9)	0.446
Vertical Transmission (Yes vs No)	−1.23 (− 11.73, 9.27)	0.817	2.99 (−8.67, 14.65)	0.613	2.62 (−7.56, 12.8)	0.611	3.87 (−6.9, 14.64)	0.478
Infection Time (≥ 20 vs < 20 years)	2.34 (− 8.53, 13.22)	0.671	−2.54 (−13.8, 8.73)	0.657	4.67 (−5.86, 15.2)	0.382	0.45 (−9.95, 10.85)	0.931
Log HBsAg	0.24 (−5.89, 6.37)	0.938	0.15 (−6.1, 6.4)	0.962	−2.02 (−7.97, 3.91)	0.501	−1.35 (−7.12, 4.43)	0.645
Log HBV DNA	−0.031 (−0.06, −0.003)	**0.032**	−3.87 (−7.08, − 0.66)	**0.019**	0.048 (0.23, 0.073)	**0.000**	−2.01 (−4.98, 0.96)	0.182
HBeAg (Positive vs Negative)	−0.57 (−8.28, 7.15)	0.885	9.48 (−3.38, 22.34)	0.147	7.79 (0.41, 15.17)	**0.039**	10.69 (−1.19, 22.56)	0.077
ALT (< 2 ULN is the reference group)								
≥ 2 & < 5 ULN	17.4 (7.66, 27.15)	**0.001**	21.4 (10.62, 32.17)	**0.000**	22.11 (13.17, 31.06)	**0.000**	22.21 (12.26, 32.16)	**0.000**
≥ 5ULN	9.89 (−2.25, 22.02)	0.110	13.41 (−0.08, 26.89)	0.051	20.89 (9.76, 32.02)	**0.000**	18.93 (6.48, 31.38)	**0.003**
Genotype (Genotype C is the reference group)								
B	−5.71 (−15.36, 3.93)	0.244	−3.93 (− 13.51, 5.65)	0.419	− 11.45 (−24.03, 1.13)	0.074	− 5.67 (− 14.52, 3.17)	0.207
N	−4.75 (− 17.88, 8.37)	0.475	−13.52 (− 29.43, 2.39)	0.095	−17.77 (− 33.46, − 2.08)	**0.027**	−11.02 (− 25.71, 3.67)	0.140
O	− 13.33 (− 29.69, 3.04)	0.110	−9.87 (− 26.29, 6.54)	0.236	−8.22 (− 17.46, 1.03)	0.081	−11.74 (− 26.89, 3.42)	0.128

Significant values are shown in boldface. B: Unstandardized Coefficients. HBV genotype: Other included C + D, B + D, B + C, D; N, not detected

*ALT* alanine aminotransferase, *HBeAg* HBV e antigen, *HBsAg* hepatitis B surface antigen;

In summary, multivariate analysis of 10 clinical-virological parameters and 10 cytokines implied that ALT was significantly associated with 8 cytokines from T cells and NK cells. Age and HBV DNA were associated with 2 cytokines, while sex was correlated with only one cytokine frequency (Supplementary Figure 2).

## Discussion

Published data indicate that adaptive responses to HBV infection are efficient and induced immediately after active virus replication begins due to the poor induction of innate immunity [14]. Other studies have shown that innate immunity may acquire a key role in dictating the course of HBV infection because of T cell impairment [24]. These controversial issues imply that a synergistic and coordinated role of all cellular components may contribute to the disease status and clinical outcome of CHB. In this scenario, our observational study involving 229 treatment-naïve CHB subjects falling in different disease phases with detailed cytokine profiles for T cells, NK cells, and NKT cells adds to the existing knowledge regarding a series of novel and important pieces of information.

We found a divergent ability of circulating T and NK cells to produce cytokines in CHB patients with different disease phases. The frequencies of half of the tested cytokines (CD4_IFN-γ^+^, CD4_TNF-α^+^, NK_IFN-γ^+^, NK_TNF-α^+^, and NKT_TNF-α^+^) were significantly higher in IA patients than in IT patients, suggesting increased inflammatory lesions in the liver of the IA patient group. Notably, the levels of other cytokines (CD8_IFN-γ^+^, CD8_TNF-α^+^, CD4_IL-2^+^, CD8_IL-2^+^, and NK_TNF-α^+^) in GZ patients were comparable to those in IA patients, implying that this proportion of patients who were not strictly recommended for treatment preserved T-cell and NK-cell cytokine functions. These findings, along with previous data from young people in the IT phase [25] reflected the inadequacy of ALT and HBV DNA levels in the assessment of disease activity [26, 27].

In agreement with previous studies [28, 29], both T and NK cells producing IFN-γ and TNF-α were positively correlated with ALT levels in the cohort of patients, especially in IA patients whose ALT expression was more than that in other groups, as well as inflammatory cytokines, highlighting a possible role of these cytokines in maintaining liver inflammation. However, no significant association of ALT with T or NK cell-produced cytokines was observed in GZ, IC and IT patients separately. Cytokines from T cells were not increased in patients with higher or lower HBV DNA levels. This association of the antiviral cytokine response with higher ALT values but not with lower HBV DNA levels might be interpreted as the rate of clearance of HBV-infected cells not being the principal determinant if steady-state HBV DNA level exists during chronic infection. Moreover, IFN-γ^+^ T cells and NKT cells had a statistically significant association with HBV DNA. The results were consistent with other reports that IFN-γ plays a prominent role in the clinical pathogenesis by recruiting inflammatory immune cells [30, 31]. Nevertheless, none of the immune cytokines produced by T and NK cells was significantly associated with HBsAg, HBeAg or other demographic characteristics of patients. A trend was seen toward a positive relationship between HBeAg level and IFN-γ^+^ and TNF-α^+^ CD4^+^ T cells in the current cohort, however, this trend did not achieve statistical significance (Table 2).

The frequency of IL-2^+^ T cells was increased in patients with genotype C although their correlation was not significant (*P* = 0.067). Genotype C has been considered to be associated with increased inflammation, high fibrosis and cirrhosis in numerous studies [32]. Thus, whether patients with higher IL-2 levels are linked to the higher clonal hepatocyte population that may lead to HCC needs to be further identified. IL-2 has essential roles in key functions of the immune system, primarily via its direct effects on T cells. However, we failed to find significant associations of IL-2^+^ with IFN-γ^+^ and TNF-α^+^ T cells in the current study. These negative data suggest that IL-2 may not be required to activate CD4^+^ and CD8^+^ T cells, whereas it may contribute to regulatory T cells (Tregs) [33–35]. A recent report demonstrated the potential of IL-2 to enhance Treg therapy in autoimmune disease [36, 37]. On the other hand, inconsistent with report of an inverse correlation found between cytokines produced by T and NK cells in CHB [24], our data showed the absence of an association regarding IFN-γ-secreting function between CD8^+^ T and NK cells. Therefore, T and NK cells may exert an additive effect to produce antiviral cytokines.

The detailed analysis of the noncytolytic control of viral infection derived from CHB patients with different disease phases included a total of 10 cytokines produced by the innate and adaptive immune response. Although at some instances significant differences emerged between the clinical-virological characteristics and cytokine profiles, it became clear that no single parameter was sufficient to distinguish the disease phases from each other based on antiviral cytokine activity. Thus, we performed correlation analysis and principal component analysis with these 10 cytokines to further improve the classification of individuals with different disease phases (Fig. 2). Using these methods, IT patients could clearly be separated from IA and IC patients. Principal component analysis also revealed that some GZ patients and the patients in the other 3 groups differed in cytokine expression. In particular, some were found to be scattered within other groups. This finding could in part explain a considerable proportion of GZ patients with high levels of inflammatory cytokines. They seemed neither to be an immune tolerate nor in an inactive state, instead, they were closer to the immune active status than to other statuses and may benefit from antiviral therapy.

## Conclusions

In summary, the current analysis takes into account clinical, virological, and immunological information collected at a single time point for each patient, and therefore the data represented only a snapshot in the long and chronic course of a disease. If liver injury mediated by the immune response is cumulative, the liver tissue injury may be related to both of the HBV specific immune response level and the infection time. Therefore, variation in infection time of the patients in the study would be a confounding factor of statistical analysis, because we detected some immunological associations in the cohort, and the bias might strengthen our conclusions; therefore, it is necessary to interpret cautiously the negative data. In this and other cohorts, the evolution of viral and immune responses and the evaluation of longitudinal disease progression could provide more information that is critical for a complete understanding of the virus-host dynamic relationship in chronic HBV infection.

## Supplementary information

**Additional file 1: Supplementary Figure 1.** Gating strategy for IFN-γ^+^, TNF-α^+^, IL-2^+^ CD4^+^ and CD8^+^ T cells, IFN-γ^+^ and TNF-α^+^ NK and NKT cells. T cells, NK cells and NKT cells were derived from total live PBMCs gated by forward and side scatter followed by single-cell gating using width and height parameters. CD4^+^ and CD8^+^ T cells were defined by the co-expression of CD3 and CD4 or CD8. NK cells were defined by the expression of CD56 and lack of CD3. NKT cells were defined by the expression of CD56 and CD3. The above cells were shown in the red boxes as indicated. The percentages of IFN-γ^+^, TNF-α^+^ and IL-2^+^ produced by these cells were further calculated according to the fluorescence of each cytokine antibody.

**Additional file 2: Supplementary Figure 2.** Summary of correlations between 10 cytokines and 10 clinical-virological characteristics was displayed as indicated. A linear regression model, Pearson correlation or Spearman correlation were used to test the correlation. *P* <  0.05 was shown in red color.

## Data Availability

The datasets used and/or analyzed during the current study are available from the corresponding author on reasonable request.

## Abbreviations

NK: Natural killer
CHB: Chronic hepatitis B
IFN-γ: Interferon-gamma
TNF-α: Tumor necrosis factor-alpha
IL-2: Interleukin-2
NKT: Natural killer T
Treg: Regulatory T cell
IA: Immune active
IT: Immune tolerant
IC: Inactive carriers
GZ: Gray zones
ALT: Alanine aminotransferase
HBV: Hepatitis B virus
MHC: Major histocompatibility complex
HBeAg: Hepatitis B e antigen
HBeAb: Hepatitis B e antibody
HBsAg: Hepatitis B surface antigen
HBsAb: Hepatitis B surface antibody
PBMCs: Peripheral blood mononuclear cells
qHBsAg: Quantitative hepatitis B surface antigen
