# Supplementary figures and images for: Association among cytokine profiles of innate and adaptive immune responses and clinical-virological features in untreated patients with chronic hepatitis B
Source: BMC Infect Dis. 2020 Jul 14;20:509. doi: 10.1186/s12879-020-05233-x (PMC7362653; doi:10.1186/s12879-020-05233-x)

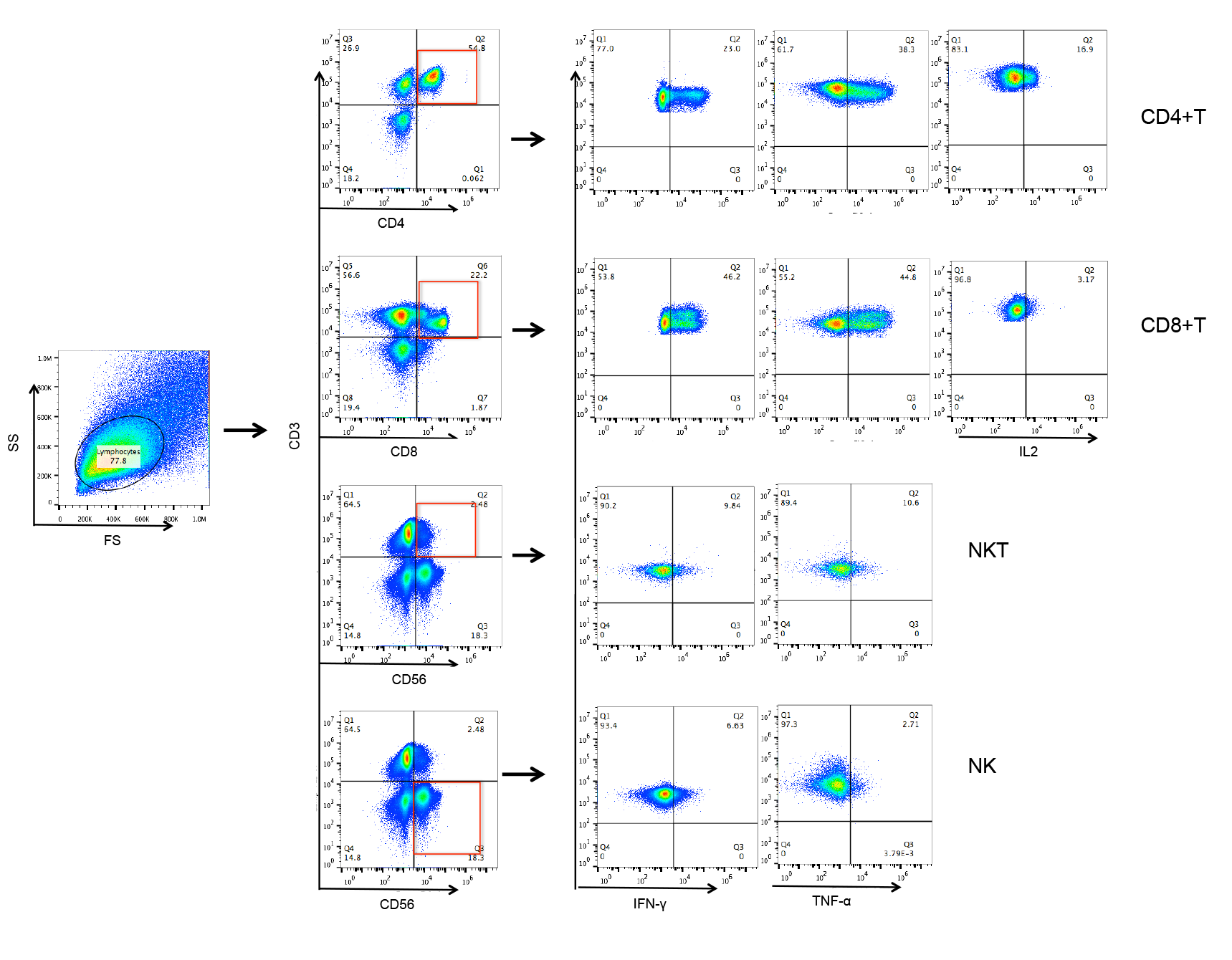

Supplement: Supplementary file 1 — Additional file 1: Supplementary Figure 1. Gating strategy for IFN-γ+, TNF-α+, IL-2+ CD4+ and CD8+ T cells, IFN-γ+ and TNF-α+ NK and NKT cells. T cells, NK cells and NKT cells were derived from total live PBMCs gated by forward and side scatter followed by single-cell gating using width and height parameters. CD4+ and CD8+ T cells were defined by the co-expression of CD3 and CD4 or CD8. NK cells were defined by the expression of CD56 and lack of CD3. NKT cells were defined by the expression of CD56 and CD3. The above cells were shown in the red boxes as indicated. The percentages of IFN-γ+, TNF-α+ and IL-2+ produced by these cells were further calculated according to the fluorescence of each cytokine antibody. [file 12879_2020_5233_MOESM1_ESM.tif]

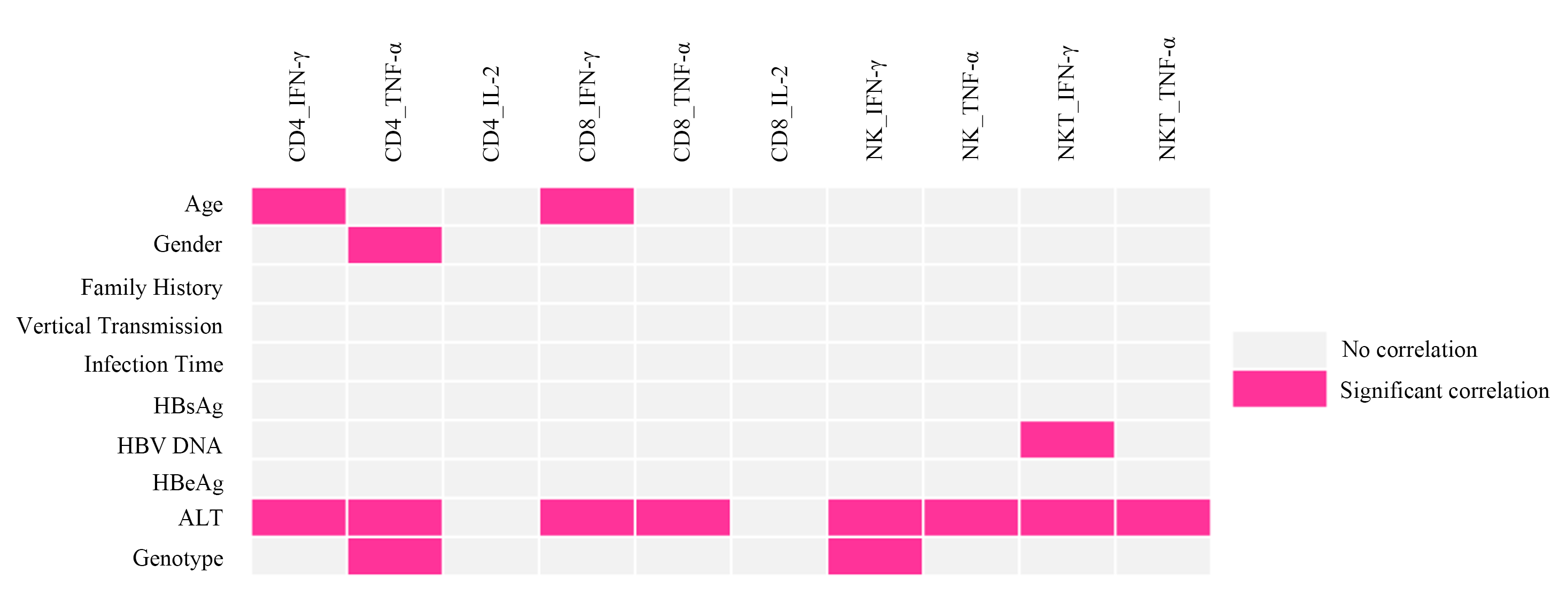

Supplement: Supplementary file 2 — Additional file 2: Supplementary Figure 2. Summary of correlations between 10 cytokines and 10 clinical-virological characteristics was displayed as indicated. A linear regression model, Pearson correlation or Spearman correlation were used to test the correlation. P < 0.05 was shown in red color. [file 12879_2020_5233_MOESM2_ESM.tif]
